# “*We have theoretical knowledge, but these are not things we do regularly*”: District hospital’s healthcare workers’ experiences and perceptions on gestational diabetes mellitus screening in Tanzania

**DOI:** 10.1371/journal.pgph.0005373

**Published:** 2025-11-07

**Authors:** Amani Kikula, Lenka Beňová, Jil Molenaar, Catherine Birabwa, Kaushik Ramaiya, José L. Peñalvo, Andrea B. Pembe, Nathanael Sirili

**Affiliations:** 1 Department of Obstetrics and Gynaecology, Muhimbili University of Health and Allied Sciences, Dar es salaam, Tanzania; 2 Department of Family Medicine and Population Health, University of Antwerp, Antwerp, Belgium; 3 Department of Public Health, Institute of Tropical Medicine, Antwerp, Belgium; 4 Department of Health Policy, Planning and Management, Makerere University School of Public Health, Kampala, Uganda; 5 Department of Internal Medicine, Shree Hindu Mandal Hospital, Dar es salaam, Tanzania; 6 Instituto de Salud Carlos III, Madrid, Spain; 7 Department of development studies, Muhimbili University of Health and Allied Sciences, Dar es salaam, Tanzania; Purdue University School of Health Sciences, UNITED STATES OF AMERICA

## Abstract

Screening for gestational diabetes mellitus (GDM) during antenatal care (ANC) is crucial for maternal and fetal health. It offers an opportunity for prevention, timely diagnosis, and treatment of cardiometabolic complications during pregnancy and over women’s and babies’ life course. We aimed to describe GDM screening service provision during ANC in two hospitals in Tanzania, focusing on gaps and opportunities for improving GDM screening services in hospitals. We employed a case-study design, with seven rounds of structured observation, two focus group discussions, and eight semi-structured in-depth interviews with healthcare workers (HCWs) from two primary healthcare level hospitals between January and April 2024. Observation notes, interviews, and discussions were audio recorded, transcribed, and analyzed using qualitative content analysis. We present our findings using three categories to describe the GDM screening services: 1) no GDM screening services were provided - screening for GDM was not conducted according to any existing national guidelines. 2) Reasons for unavailability of GDM screening services - there was inadequate support for GDM screening services at the hospitals: no continuous learning opportunities for HCWs on GDM screening, a shortage of screening supplies, and the hospitals did not prioritize GDM screening services. 3) Opportunities for improving GDM screening services - the inclusion of GDM in the structural and administrative agenda of the hospitals was deemed important to provide quality screening for GDM. There was a discrepancy between guidelines and actual GDM screening services at the hospitals studied. To provide GDM screening services per the available national guidelines, HCWs should be trained and supported with supplies for providing GDM screening services.

## Introduction

Women continue to face preventable pregnancy and childbirth-related complications, with most maternal deaths occurring in sub-Saharan Africa (SSA) [1]. While Central and Southern Asian regions made strides towards attaining the 2030 Sustainable Development Goal 3.1 of reducing their maternal mortality ratio (MMR) to less than 140 deaths per 100,000 livebirths, the sub-Saharan region made little progress in reducing their MMR between 2000–2020. [1]. Tanzania recorded a substantial reduction in MMR from 556 in 2015/16–104 MMR in 2022 [2]. However, indirect causes of maternal deaths, predominantly non-communicable diseases such as diabetes mellitus, constitute a rising contribution to maternal mortality and morbidity and pose a significant challenge to further reduce MMR [3–6]. This obstetric transition is causing a double burden of direct and indirect causes of maternal deaths, complicating efforts put in place to address preventable maternal deaths [7,8].

The global population is experiencing an epidemiological transition towards increased obesity, and in Tanzania, like in most low and middle-income countries, this transition is seen also in women of reproductive age [9–12]. Obesity is strongly correlated to the development of cardiometabolic conditions, including but not limited to diabetes mellitus [13]. When diabetes is related to pregnancy, either as first onset or first diagnosed during pregnancy, it is termed gestational diabetes mellitus (GDM) [14]. GDM increases women’s risk of hypertensive disorders, intrauterine fetal death, operative delivery, and birth trauma. Long-term consequences include an elevated lifelong risk of developing diabetes mellitus and other cardiometabolic conditions. For the newborn baby, complications include macrosomia, birth injuries, respiratory distress, and hematological complications [15]. In utero exposure to maternal hyperglycemia because of GDM predisposes children to an increased risk of adult obesity and diabetes mellitus [16]. Timely screening for GDM offers a window of opportunity for prevention, diagnosis, and treatment of cardiometabolic conditions including GDM, not only during pregnancy but for the life course of the women and newborns [16–20].

Screening services during pregnancy aim to prevent, diagnose, and treat multiple conditions to help foster a positive pregnancy experience [21,22]. In Tanzania, the Ministry of Health (MoH) provides guidelines for GDM screening and diagnosis, health education in general, and for care strategies following a GDM diagnosis [21,23]. Tanzania has two guidelines for GDM, each recommending a different modality for GDM screening. Despite having two guidelines in place, little is known about what is implemented, gaps in implementation or the existing opportunities for improvement of GDM screening services in Tanzania. In Tanzania there is no nationally-representative GDM prevalence estimate, but facility-based studies in both rural and urban settings have reported GDM prevalence ranging from 4.3% to 39% [24–27].

Primary healthcare level facilities are the entry point to healthcare for most Tanzanians, including women seeking ANC services [2,28,29]. Optimal implementation of GDM screening guidelines within the Tanzanian context has the potential to reduce GDM’s contribution to national maternal and perinatal morbidity and mortality rates. Therefore, this study aimed to qualitatively describe the screening practice, gaps, and opportunities for improving GDM screening services in two primary health care level hospitals from a healthcare workers’ (HCWs) perspective.

## Materials and methods

### Study design

We employed a case-study design to analyze GDM screening service within two hospital-based ANC clinics, utilizing structured observations, focus group discussions (FGD), and in-depth interviews (IDIs). For this study, we considered GDM screening service within a hospital-based ANC clinic as a case.

### Study context

This study was conducted in two public primary health care hospitals at the district hospital level in Tanzania. In Tanzania, the primary health care level is composed of the community health services, dispensaries, health centers, and district hospitals at the top of the primary health care hierarchy. After the district hospitals, there are regional, zonal, and national hospitals [30]. Hospital A is located in a rural district of the Coast (Pwani) region and Hospital B is in a semi-urban district of Dar es Salaam region. In 2022, the Coast (Pwani) region had a population of over two million people [31] while Dar es Salaam had over five million people [32].

Both hospitals serve as referral hospitals in their respective districts. Obstetrics and gynaecology departments in both hospitals have an in-patient and an out-patient unit. The ANC clinics are within the outpatient unit and comprise routine ANC clinics (managed by nurse-midwives and general practitioners) and specialized clinics (managed by medical specialists). To provide ANC services, the obstetrics and gynaecology department co-functions with the laboratory, radiology, and reception departments. A summary of relevant hospital characteristics is presented in Table 1.

**Table 1:** Characteristics of study hospitals related to the provision of ANC services.

Characteristic	Hospital A rural district of Coast region	Hospital B semi-urban district of Dar es Salaam region
Annual ANC attendance	5,000 women	14,000 women
Organization of care and ANC staffing	Specialized clinic (one day per week): three full-time obstetricians	Specialized clinic (two days per week): two full-time obstetricians
	ANC clinic (weekdays): eight nurse-midwives and two clinical officers.	ANC clinic (weekdays): six nurse-midwives and two clinical officers.
Laboratory capacity	Functioning laboratory that serves the entire hospital with 14 full-time staff. ANC-specific tests: full blood picture, blood grouping and cross-matching, hemoglobin level, urinalysis, syphilis test, stool analysis, blood glucose test, malaria blood test.	Functioning laboratory that serves the entire hospital with 7 full-time employed staff. ANC-specific tests: full blood picture, blood grouping and cross-matching, hemoglobin level, urinalysis, syphilis test, stool analysis, blood glucose test, malaria blood test, hepatitis test.

Women attending ANC clinics at these hospitals are routinely seen by nurse-midwives, or by doctors if they have an underlying medical condition or had been referred. The care provided is based on the MoH ANC guidelines (further referred to as “the ANC guideline”) and the Tanzanian standard treatment guideline (further referred to as “STG”) [21,23]. The ANC guideline recommends preventive measures for several conditions of public health importance. During ANC visits, women are supposed to receive group health education on multiple topics including: birth preparedness, emergency pregnancy symptoms, nutrition, and non-communicable diseases. Additionally, the guidelines recommend prophylaxis for malaria, hookworm infestation, and anemia, as well as screening/testing for and management of conditions such as HIV and syphilis, urinary tract infections, hypertension, and diabetes.

The two guidelines present divergent approaches to screening for GDM. The STG [23] recommends a risk-based screening strategy, employing a checklist during women’s first ANC visit to identify women at high risk of GDM (any of the following: history of GDM, previous big baby, poor obstetric history, family history of DM, known impaired glucose tolerance/impaired fasting glucose, grand multipara, glycosuria or BMI > 25 kg/m^2^) who should undergo a 75-gram oral glucose tolerance test (OGTT) at 24–28 weeks. On the other hand, the ANC guideline recommends routine glycosuria testing as an initial test to identify women at high risk for GDM, and those with glucose in urine will further be tested with a random blood glucose test [21].

In terms of health education, only the ANC guideline instructs on how health education should be provided. The ANC guideline instructs on the provision of reading materials, video shows, health education talks for women attending ANC clinics, individually or in a group. The topics include 1) self-care, gender-based violence, hygiene and substance use, 2) birth, danger signs and emergency preparedness, 3) tests to be done during ANC, 4) prevention of HIV, malaria, diabetes and other diseases, 5) nutrition and physical activities 6) supplementation and vaccination during pregnancy, 7) family planning, newborn and postpartum care, and 8) work, sexual activity, rest, travel and medication use.

In this study, we define “GDM screening services” as group health education related to GDM, nutritional counseling and GDM screening tests. This is to align with the patient-centered approach of the ANC guideline where, apart from the tests done, group health education should be provided as part of ANC to encourage women’s understanding of the need for GDM screening tests during pregnancy.

### Population and sample

We purposively selected two hospitals in a primary health care setting to understand GDM screening services based on volume of the ANC attendees, having at least a full-time gynecologist and being located in a rural area and an urban area. Furthermore, we needed a hospital with a high volume of clients receiving ANC (≽ 10,000 per year), one with low volume (≼ 5000).For the structured observation, we included all hospital departments and HCWs involved in providing and supporting ANC services and their interactions with pregnant women. We focused our observations on the activities and interactions in three key areas of the hospital: 1) reception/registration area where women coming for ANC visits need to register, 2) laboratory HCWs, and 3) ANC clinic HCWs.

We conducted two FGDs, one in each hospital, involving nurse-midwives stationed at the ANC clinics. Hospital A’s FGD had seven participants, while Hospital B’s had five participants.

For the IDIs, we purposively selected eight participants based on their experience and daily responsibilities related to ANC service provision. The IDI participants included three doctors, two laboratory staff, and three nurse-midwives, among them, two were participants of the FGD. Participants from both hospitals were interviewed (Table 2).

**Table 2:** Characteristics of IDI (n = 8) and FGD participants from the study hospitals (n = 12).

Characteristic	Number of participants
Gender	
Male	7
Female	11
Age	
20-29	2
30-39	7
40-49	9
Cadre	
Nurse-midwives	12
Doctor	4
Laboratory officer	2
Hospital	
A	10
B	8

### Data collection

#### Structured observation and assessment.

The first author (AK) conducted seven rounds of observations in the two study hospitals, totaling 192 hours. Different operational periods were strategically captured, including both morning and afternoon hours, busy and relatively less busy ANC clinic days of the week. Key focus areas of observation included the physical setup (ANC clinic); patient flow (movement of women throughout their visits); service delivery (how women receive care and by whom); laboratory screening procedures (types of tests conducted); and health education (availability of informational posters and provision of group health education services). A structured facility assessment checklist was conducted during the first round of observation, assessing 1) HCWs availability for ANC and laboratory services, 2) guideline availability and accessibility to all ANC HCWs, 3) availability and functionality of laboratory tests relevant to ANC services, and 4) service provision for women coming for ANC.

**Fig 1:**
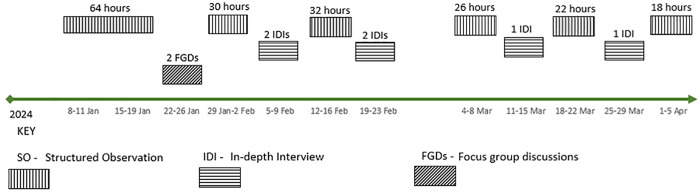
Flow of the data collection process, by calendar week.

The first round of observation (64 hours) which covered all areas of interest in the provision of GDM services, preceded the FGDs and IDIs. This allowed AK to better understand what and how GDM screening services were delivered, which were further explored in the FGDs and IDIs. Next, two FGDs were conducted (one in each hospital), which provided an insight into a collective description of elements of GDM screening services provided. Subsequently, rounds of IDIs alternated with rounds of observations. This allowed for iterative adaptation of the observation checklist and IDI guides to understand the nature of GDM screening services provided at the two hospitals and reasons for discrepancies between observations and IDIs/FGDs. Fig 1 shows the order of the data collection methods during the study. Detailed field notes were documented daily for preliminary analysis and these informed concurrent adaptations of the interview guide.

#### Focus group discussions and in-depth interviews.

AK conducted the IDIs and FGDs with HCWs within hospital premises in locations that ensured the privacy of the conversations supported by a research assistant who took notes. These included offices, clinic/consultation rooms, and seminar rooms. IDIs were scheduled with participants to accommodate the time with no active clinic/hospital activities or outside working hours. Topics covered in the IDI and FGD guides included the ANC services provided to women, the practices related to group health education in general and on GDM, the practices for GDM screening, and possible underlying factors shaping the existing practice. Additionally, using the topic guide we explored the availability of existing forums for continuous medical education (CME) of HCWs, as well as their recommendations to improve GDM screening services and laboratory support related to GDM. The tools (S1 Text), assessment checklist (S2 Text) and observation guide (S3 Text) can be found in the respective supporting information files.

The IDIs and FGDs were conducted in Swahili, recorded, and transcribed verbatim then translated to English. Data collection took place over a period of 13 weeks, from January to April 2024 (Fig 1).

### Data analysis

Iterative triangulation of information from all three data collection methods guided the point of data saturation. Saturation was considered when similar repeated responses on views about the questions asked were expressed by participants in both hospitals. In addition, prolonged engagement and observation supplemented the recognition of the point of data saturation [33,34] through triangulating what was observed with what was shared in the interviews and discussions. Discussions with NS and ABP during data collection enabled collective interpretation of the topics covered, research questions answered, and areas for further exploration.

AK familiarized himself with the transcripts and field notes of the whole data set through reading and re-reading the transcripts. All transcripts were then reduced to meaningful units and further to open codes. These were further inductively reduced to codes. AK selected three rich transcripts and shared them with JM and CB for independent coding which was followed by a discussion for code triangulation and consensus about the final coding tree. The coding tree was used to objectively code transcripts and notes from the three data collection methods in line with the study objectives using qualitative content analysis, inspired by Graneheim and Lundman [35] using NVivo 1.7.1 (1534) software. AK and NS grouped the codes into sub-categories and then the final three categories.

AK, NS, and ABP are fluent in Swahili and English; JM is fluent in English and conversant in Swahili, while CB is fluent in English. AK did the coding in Swahili and translated the codes to English, JM and CB did the coding using English transcripts; the code triangulation was done in English. JM and AK reviewed all selected quotes to ensure accurate translation from Swahili to English.

### Ethical considerations

The study received ethical approval from the Institutional Ethics Committee at the Institute of Tropical Medicine, Antwerp 1687/23, Muhimbili University of Health and Allied Sciences Research and Ethics Committee MUHAS-REC-07-2023-1834 and the National Health Research Ethics Review Committee NIMR/HQ/R.8a/Vol.IX/4457 (NatHREC). A research permit was also received from the President’s Office, Regional Administration and Local Government, research unit AB.307/223/01, regional offices (Dar es Salaam and Coast region), District Health offices and the hospitals. The hospitals’ medical officers in charge provided permission for observations and the study’s observational data collection methods were clearly explained to HCWs. Written informed consent was obtained from all interviews and FGD participants. All transcripts were de-identified before analysis. We chose to keep the hospital names anonymous to safeguard the confidentiality of the participants.

## Results

Overall, GDM screening services in the two study hospitals were not provided in line with the two existing national guidelines. We identified three major categories within the data: 1) no GDM screening services provided as per national guidelines 2) reasons for unavailability of GDM screening services and 3) opportunities for improving GDM screening services.

**Fig 2:**
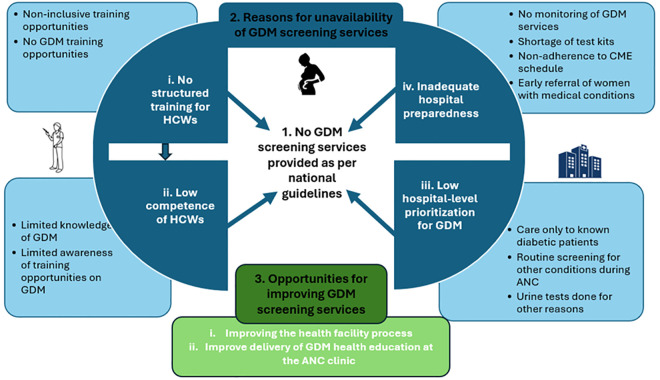
Description of the results using categories, sub-categories and codes.

Fig 2 shows the build-up of the three categories from the code level and linkages between the categories. Neither hospital provided recommended GDM screening services as per national guidelines. In both hospitals, GDM screening was not carried out and GDM was not included in ANC group health education. GDM screening services were unavailable due to four main factors: 1) lack of structured training on GDM for HCWs, which contributed to 2) HCWs feeling insufficiently confident in their ability to conduct GDM screening. Additionally, 3) hospitals did not prioritize GDM services, and 4) hospitals were not adequately equipped to deliver GDM screening services.

1. No GDM screening services are provided as per national guidelines

In the two study hospitals, GDM screening services, including group health education and screening tests, were not provided as recommended by the national guidelines.

### Unstructured group health education.

In hospital A, HCWs reported performing daily group health education sessions at the ANC clinic, “…*in the clinic here, health education is provided every day* …” (Doctor 1, IDI). In hospital B, these sessions were only consistently provided on Tuesdays, as noted by a nurse-midwife: “…*if you come on a Tuesday, you will see the sessions being conducted…*” (Nurse-midwife 2, FGD). This day was reserved for pregnant women coming for the first ANC visit. This was consistent with what was observed during the study period. Furthermore, in both hospitals, group health education sessions were conducted only in the early morning (between 07:30–09:00 am), which consistently missed women who arrived at the clinic later.

The lack of standardization in group health education content and scheduling was evident. Consequently, HCWs prioritized topics based on the perceived immediate needs of the ANC attendees and focused on the conditions they encountered frequently: *“…anemia has become a constant problem, so in the topics, we often talk about anemia…”* (Doctor 2, IDI). This resulted in other key topics and conditions, such as GDM, being overlooked. Another HCW highlighted how a knowledge gap meant HCWs were not comfortable delivering health education sessions on GDM: “*No, I don’t think we’re comfortable even speaking about it, as I said earlier, we haven’t taken it seriously to understand it…, and diabetes is not discussed in those topics that women are given in health education sessions…”* (Doctor 2, IDI).

Observed health education sessions primarily focused on emergency and birth preparedness, with occasional inclusion of elements from specific ongoing outbreaks or health campaigns. However, diabetes during pregnancy was never included in the education session topics provided in the ANC clinic in both hospitals: “*Honestly, no, we have never discussed about diabetes in pregnancy in the health education sessions...” (*Nurse-midwife 1, IDI). We observed that each hospital had a monthly content guide for providing group health education content. This was prepared at the beginning of the year by the ANC nurses, however, none of these had GDM as one of the topics.

**Fig 3:**
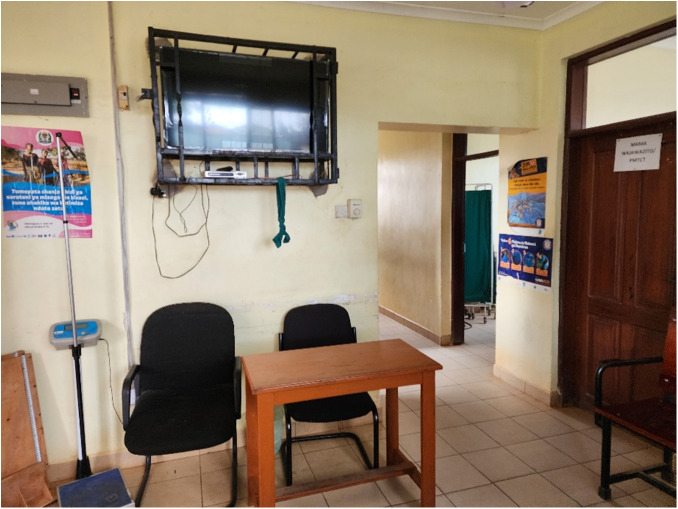
Setup of a women waiting area for those attending for ANC showing the presence of un-used TV.

In both hospitals, some resources were underutilized for providing health education in general. Both hospitals had television screens in the ANC clinic (Fig 3) which were observed to be unused during the data collection period. These could potentially serve as platforms for delivering standardized, already available MoH-approved digital educational materials to women waiting for ANC services.

### Sub-optimal use of GDM screening tests.

Similarly, the GDM screening tests (checklist screening test, glycosuria test and OGTT) were not routinely conducted in the studied hospitals according to either of the MoH guidelines. As one HCW noted, “… *it is not a routine test, like the other tests, like VDRL (syphilis test), PMTCT (HIV test) or Hb (haemoglobin test)* …”(Doctor 2, IDI).

During data collection, we also focused on how HCWs utilized pregnant women’s urinalysis results. This routine test, performed during ANC visits, is endorsed by both the STG and ANC guidelines of Tanzania as a screening tool for GDM, urinary tract infections, and proteinuria. However, the rationale for doing it in these hospitals was mainly for checking protein in urine or infection but not glucose in urine. The other two screening tests - OGTT and checklist screening test - were not done in either hospital: “…*since I started working here, no, the glucose tolerance test has never been done…*”(Doctor 1, IDI). This was further confirmed during observation by the absence of the screening checklist and OGTT solutions at the clinics and hospital laboratories.

Even though some HCWs were aware of the existence of the guideline/s that described screening tests for GDM, the performance of the GDM screening tests was only done occasionally by a few HCWs from the ANC clinic. This observation was further confirmed by the laboratory staff, noting the small number of blood glucose test requests from the ANC clinic compared to other hospital departments *“…it’s very low for diabetes test..*.” (Laboratory personnel 1, IDI) which aligned with the information from observations and from interviews. The observed practice demonstrated a clear prioritization of other conditions over GDM during ANC, as underscored by the stark contrast between the urgency given to hypertensive disorders over other NCDs, including GDM, in ANC practices. It was also visually evident in the ANC clinic and adjoining patient waiting areas, where available health education posters predominantly focused on emergency signs during pregnancy, malaria and HIV/AIDS, with no representation of diabetes.

“*Blood pressure in pregnancy is discussed a lot more and given major priority, maybe because it was seen as a major contributor to maternal and neonatal deaths? Because even before we start our services, we start with checking weight and blood pressure, then the rest follows… If it’s high, even before I provide you with routine ANC care checks, you are given medication while we wait for it to lower down. But regarding diabetes, we still don’t do anything about it…”* (Nurse-midwife 1, IDI)

2. Reasons for unavailability of GDM screening services

### No structured training and low competency of healthcare workers on GDM.

Among the points which HCWs mentioned was their insufficient knowledge on GDM: “*Currently, the knowledge on diabetes is lacking among our colleagues in the healthcare field...”* (Doctor 1, IDI). This knowledge gap affected HCWs’ ability and competence to provide GDM-specific group health education and conduct recommended screening tests. The absence of GDM-focused in-service training within the two hospitals contributed significantly to this knowledge gap, as highlighted by a HCW: “…*I’ve never heard of a training on GDM, no one has. Because I’ve been working with pregnant women for a long time…*”(Nurse-midwife 3, IDI). The existing in-service trainings offered to the HCWs were predominantly focused on HIV, malaria, and emergency obstetric care; further widening the knowledge gap with GDM. These trainings were either hosted within the hospital or outside the hospital and organized by different health stakeholders. In addition, at the hospital departmental level, fewer ANC HCWs received any available training opportunities offered compared to HCWs from other units within the department (e.g., the labor ward staff) “…*but unfortunately, it’s usually those who are stationed in the labor ward who are the ones who attend the trainings most of the time* …” (Doctor 2, IDI).

The hospitals also offered internal CME sessions alongside external training opportunities. These were intended to involve the HCWs of the whole hospital or within respective various departments. These in-house CME sessions lacked consistent scheduling and did not typically meet their educational objectives. These internal CME sessions, if properly structured, could serve as useful platforms for enhancing healthcare workers’ knowledge, including their understanding of GDM management.

“…*There is a training schedule, but it’s mostly not implementable; I mean, it exists in the schedule, but implementation can be difficult. Continuous medical education sessions are supposed to be given every Wednesday, involving all departments. However, Wednesdays pass without training; sometimes it’s just a regular meeting*…”(Nurse-midwife 2, FGD)

### Low hospital-level prioritization for GDM services.

In general, at the hospital level, HCWs perceived that GDM was less prioritized as compared to other health services provided *“…we can say it has been overlooked in our hospital, and we’ve been taking it as business as usual…*” (Doctor 2, IDI). This has limited the preventive services provided during ANC to only a few conditions, rather than providing a comprehensive package that covers all conditions as recommended by the guidelines. On an even higher level, the specialists pointed out that even during their specialization training, GDM was not something that was emphasized *“…I think maybe we’ve taken this issue of diabetes in pregnant women as a norm because even during our training when we used to go for those community services, I don’t think we were screening for it…”* (Doctor 2, IDI). This negatively affected every aspect of care related to screening for GDM, where HCWs in the unit and even the ones supervising them (specialists) were not conversant on the condition leading to a collective lack of confidence in providing GDM care according to guidelines.

“…*No, I wouldn’t be comfortable unless I revise on it, because not everyone listening to health education is totally ignorant; they know. So, as a service provider, I’ll provide health education about diabetes, but then, when I reach certain points and stumble, they might think, why are they teaching us while they are not even conversant on the subject? So, there’s a need to revise more so that we get updated on the subject so that we can explain to women for them to understand because these are not things we do regularly; we have theoretical knowledge, but these are not things we do regularly…”* (Nurse-midwife 1, IDI)

In terms of group health education sessions, HCWs pointed out that they were not aware of a guideline that could be used to plan for such sessions “*There isn’t any guideline... I don’t think there’s a direct guideline...”* (Doctor 2, IDI). This resulted in a situation where HCWs were more competent in addressing certain pregnancy conditions and not addressing other conditions: *“Malaria is well understood, so we have some ideas. Hypertension because of many eclampsia patients, eclampsia, it’s also talked about a lot, more than diabetes for pregnant women…”(*Nurse-midwife 3, IDI)

The researcher observed that within the ANC clinic, there was consistent follow-up and supportive supervision on quality of data reporting for other preventive services offered during ANC such as HIV screening, malaria prophylaxis, anemia, deworming, and blood pressure measurement. There was, however, no such initiatives given to GDM screening services.

### Lack of hospital preparedness to offer GDM screening services

Moving towards the operationalization of the day-to-day activities, the laboratories in both hospitals faced challenges in providing consistent GDM screening services due to their partial reliance on support from donor-funded activities. These were ongoing programs within the bigger context of routine hospital activities, particularly HIV-related programs. The programs supported HIV testing and treatment: “…*they support patients with HIV, … even mothers with HIV … they support them.”* (Laboratory personnel 1, IDI). This included laboratory supplies meant to facilitate the performance of the tests. These tests were at times useful even for HIV-negative pregnant women. This support to hospital laboratories trickled down to other services (e.g., full blood picture reagents), which reduced the hospital expenditure for some laboratory supplies needed during ANC. However, this reliance on donor funding led to unstable supply chains, particularly during periods of limited donor support. The resulting resource constraints forced hospitals to prioritize certain supplies over others, leading to stock shortages of some test kits. Consequently, instead of following guidelines recommending universal screening, healthcare workers had to ration tests, offering them only to patients deemed high-risk.

“…*I mean, when you order two or three, four people, they (the lab) get shocked and say, “choose those who really need it” (mmmh), those in need because if you start testing everyone, that kit (glucose test kit) with its strips you know it runs out quickly…”* (Nurse-midwife 3, IDI)

In hospital B, there was limited space to admit many patients and limited laboratory capacity for some tests that can be used to monitor women with other medical conditions like diabetes. This resulted in the practice of early referral of most pregnant women with or suspected to have medical conditions to higher-level hospitals. This created a vacuum of skills to take care of such patients and created a sense of low prevalence of some diseases, including GDM, because they are not seeing the outcomes of such patients “…*It’s possible there were others I encountered but because I don’t see them right to the end of delivery*…” (Doctor 3, IDI). The other component was the mismatch between the presence of guidelines for these hospitals and their accessibility as a clinical reference tool to be used as intended. This was observed, for example, by the absence of a physical copy of the guidelines in one of the ANC clinics and reported in IDIs in a hospital where a copy of the guideline was found locked in the cupboard while the HCW was unaware of its existence.

“*…honestly, on my side, let me respond from my end. There may be one [guideline], but I haven’t seen it. … I’ll give my personal know-how. It may exist, but I personally haven’t seen it…”*(Nurse-midwife 2, IDI)

3. Opportunities for improving GDM screening services

#### Improving the health facility processes.

HCWs offered a number of recommendations on how GDM screening services can be better structured in their hospitals. Firstly, HCWs emphasized the importance of involving the hospital administration in institutionalizing GDM screening services within routine services offered “…*because they need to understand for them to see the need to increase the budget for equipment and reagents; administration needs to be aware…”* (Doctor 2, IDI). This engagement is crucial for securing and sustaining finances for GDM screening and management.

HCWs agreed that all women should be screened for GDM and underscored the need to find a stable financing modality to ensure that screening for GDM is provided equitably for all women. However, they worried about where the finances would be found for this, and they were concerned about the impact of introducing an out-of-pocket payment for women without health insurance. The efforts to incorporate GDM screening into routine ANC services should go hand in hand with the need to develop a comprehensive package of required tests for pregnant women, which they said should include routine blood glucose testing. They further suggested that all rapid tests, including those for GDM, be conducted at the ANC clinic – rather than at the laboratory, similarly to how HIV rapid tests are done. One nurse-midwife articulated this idea: *“...we can simplify it, test them here, give us the machines with those strips, we can do it here, rather than them coming and you want to test them, then they have to go back to the lab...*” (Nurse-midwife 2, FGD). This approach would streamline the process and reduce the complexity and duration of women’s ANC journeys in the hospital.

#### Improve delivery of GDM health education at ANC clinic.

Furthermore, HCWs highlighted the importance of developing information, education, and communication (IEC) materials for both women and healthcare workers. As noted by a participant, “*The poster and brochures will help, when someone is giving education, they see each aspect there on one brochure too..*.” (Nurse-midwife 3, IDI). These materials could improve routine group health education session delivery and understanding of GDM among ANC attendees.

Providing education related to GDM to women attending ANC was also deemed essential in improving compliance and creating demand for GDM screening services. One participant remarked, *“...the nature of the women we attend to here, once they understand, they have no problem...*” (Nurse-midwife 1, IDI). To ensure they are competent to provide the required health education on GDM, HCWs stressed the need for continuous education for themselves. They suggested utilizing internal hospital CME sessions or inviting experts to train them. They also suggested having more GDM-related training opportunities for the HCWs to improve their confidence and competence in caring for GDM. As one participant explained,

*“...the need is greater than what you think because you will always find patients with diabetes being pushed to one doctor, not because they specialized in it, but mostly it’s because they had a one or two-weeks training, so they’ve been slightly exposed to care on diabetes, meaning people might lack the confidence to care for diabetic clients, so the need is great...*” (Nurse-midwife 1, IDI).

## Discussion

In this study we describe the extent of GDM screening services provided at the ANC clinics of two hospitals at the primary healthcare level in Tanzania. In these hospitals, GDM screening was not routinely done as per national guidelines and GDM was not a topic within the group health education sessions provided to ANC attendees. Healthcare workers identified several systemic barriers to effective GDM screening, primarily the absence of a comprehensive CME framework. The lack of GDM-specific training opportunities, combined with minimal financial allocation due to low prioritization, hampered GDM service delivery. Furthermore, the screening system demonstrated a bias toward communicable diseases, leaving GDM largely overlooked despite existing guidelines. The HCWs recommended an improved hospital system that includes training of HCWs on GDM, ensuring sustainable availability of supplies for delivering GDM screening services, and sensitizing the hospital administrators and community on GDM.

The sub-optimal screening services for GDM in ANC observed in these two hospitals echoes previous reports from hospitals across Tanzania [24,26,28]. Even in health facilities with sufficient laboratory capacity to carry out the recommended tests, routine screening is not always performed [28,36]. This could be due to the vertical nature of the programming of most services that ends in fragmentation and prioritization of other, better-known services such as HIV within ANC clinics [37]. As seen in our results, other components that facilitate the delivery of services including formal or informal training and financing become biased toward such systemically prioritized services. The exclusion of GDM as a key performance indicator of the ANC services [21] has inadvertently reduced its monitoring priority compared to other ANC indicators. It is imperative to consider ANC as a single operational unit rather than fragmenting it based on the services provided. Borrowing from the bundling of the clinical treatment models for different emergency interventions [38,39], ANC screening services could benefit when bundled as point-of-care tests in a similar way. This will save time that women use at different points for different tests but also ensure none of the screening tests are missed.

At the ANC clinic where multiple services are expected to be offered, clinical guidelines are meant to provide directions to the HCWs for how all the services are to be provided. For GDM, where multiple screening algorithms are in place, its practical translation to routine services can be limited [21,23]. The lack of proper integration of GDM care guideline recommendations within ANC is also observed in most other African countries [40,41]. Further in South Africa, fragmentation of health care services have left the less financed services un-attended to, including diabetes care within ANC services [42]. In Europe, due to existence of multiple international guidelines on GDM care, it has led to partial implementation into daily clinical practice due to conflicting evidence [43,44]. From our results, while majority of the HCWs narrated being unaware of the guidelines on GDM, it was not pointed out to be the reason for not implementing GDM screening services. Participants narrated on lack of general knowledge on GDM, overlooking the guidelines available. This underscores the need for having and developing guidelines that are used by HCWs rather than those that stay in office cabinets [45].

Successful translation of these guidelines into practice requires a supportive health system that positions healthcare workers at the center of implementation, providing them with adequate training, comprehensive monitoring systems, and sustainable resources [46]. The hospital system should also promote within and inter-hospital multidisciplinary collaboration for services that involve multiple departments [47,48]. This challenges the top-down introduction approach of clinical guidelines with practice of printing and distribution of the guidelines. Rather it should be aiming at having guidelines with clear inclusive aims, functional financing and checking mechanisms while accommodating the contextual differences on where it is to be implemented with the HCWs at the center of it all [48–50].

Receiving essential services during an ANC clinic visit is a proxy indicator for quality ANC services [51]. In Tanzania, less than half (40.9%) of women received adequate ANC services [52]. The services that require relatively less contact time with the client, including blood test and checking the baby’s heartbeat, are offered to most women (over 90%) [52]. For services like counselling, which over two-thirds of ANC attendees receive [2], the quality of counseling is substandard and do not cover all topics [53] similarly to what was observed in this study with the exclusion of GDM topic during group health education sessions. In the same ANC clinic setting, HCWs are commonly overwhelmed with multiple tasks necessitating multitasking and task shifting due to staff shortage [54,55]. As a result, the overall quality of care is compromised [56], particularly in service components that require prolonged engagement or those that receive insufficient prioritization and resources. It is imperative to recognize time as a critical resource in the delivery of quality ANC services.

While Tanzania has made remarkable strides in HIV screening during ANC, achieving a 98% testing rate among ANC attendees in 2023 [57], this success presents an opportunity to extend similar efforts to other preventive maternal health services. The effectiveness of funding, monitoring, and capacity-building strategies for HIV screening could serve as a model for integrating additional routine services, such as diabetes screening, within ANC [58]. Extending these lessons to GDM care in ANC would potentially translate to the prevention of undetected diabetes related complications during pregnancy and the life course of the woman and the newborn [16,19]. By viewing ANC as a holistic platform, stakeholders can strengthen systems to implement all recommended services outlined in the guidelines. This approach not only engages all relevant actors but also builds resilience, allowing the system to adapt to future needs and innovations, ultimately contributing to a positive pregnancy experience [59].

### Strengths and limitations

Through the use of interviews, focus group discussions, and observations, we iteratively triangulated information derived from either data collection technique to better understand GDM screening services offered at the two hospitals. Continuous discussion among the study team members during data collection, and independent coding with researchers of different backgrounds allowed objective interpretation of the data collected. We acknowledge that our findings were derived from two public district hospitals that have a different organizational and operational composition from private hospitals or higher-level hospitals. We also acknowledge not having hospital administrative inputs on GDM screening services offered. However, we believe these findings reflect what was practiced in the hospitals. The findings highlight the need for improving the hospital practices that will allow improvement in GDM screening services in these hospitals and settings with similar context. Future exploration of how the district and hospital health management teams can influence the realization of GDM screening services will be crucial in having routine GDM screening within the ANC clinic. This is because HCWs are part of the health system and work within an environment that is governed and influenced by the health administrators [60,61].

## Conclusion

GDM screening services were not routinely offered in the two study hospitals. The existing health system did not support HCWs within the hospital to screen for GDM in ANC clinics as national guidelines. For GDM screening to be optimally performed, all stakeholders should be engaged, HCWs trained, and work in a supportive environment to provide care according to existing guidelines.

## Supporting information

S1 Text Topic guides for IDI and FGD. (PDF)

S2 Text Hospital assessment checklist. (PDF)

S3 Text Structured observation guide. (PDF)
