# Supplementary material for: “We have theoretical knowledge, but these are not things we do regularly”: District hospital’s healthcare workers’ experiences and perceptions on gestational diabetes mellitus screening in Tanzania
Source: PLOS Glob Public Health. 2025 Nov 7;5(11):e0005373. doi: 10.1371/journal.pgph.0005373 (PMC12594363; doi:10.1371/journal.pgph.0005373)
Supplement: S1 Text — (PDF) [file pgph.0005373.s001.pdf]

Version 3 (25/01/2024)

Optimizing screening practice for gestational diabetes mellitus in primary healthcare facilities in Tanzania

S1A appendix: Topic guide for FGD

Group discussion Metadata								
	P1	P2	P3	P4	P5	P6	P7	P8
Age								
Sex								
Marital status								
Highest level of education attained								
Cadre								
Languages spoken								
Position (work station)								
Date of the FGD								
Place of interview								
Start time								
End time								

1. Good morning everyone, we are here to have a conversation about the services that a woman receives when she comes for ANC, particularly on screening for diabetes during pregnancy. Can we have someone tell us, when a woman comes-in to the point of exit where and what she does on a routine clinic visit.

Optional prompts for other members if not mentioned:

a. Which entrance does she use
b. Where does she register
c. The time that the clinic starts
d. Which service is offered first
e. Does she pay for any service?
f. How is she positioned to be attended in the order of her arrival to the clinic (cards collected/numbers given or what is done?
g. Who is likely to see her for a consultation (doctor or nurse or both and what will make the difference)
h. How/where does she get a date for next visit

2. Then can we now talk about the screening modalities. In this facility, can someone tell us how do we identify if a woman has diabetes during pregnancy

Optional prompts for other members if not mentioned:

a. Do we screen for GDM? If yes to all?
b. Which guideline are we using.
c. What are the reasons to choose the modality we have chosen to use.
d. If no screening done, why are we not doing? Are there challenges to do screening routinely?
e. Are we aware of Tanzanian guideline/s that are speaking about screening for diabetes in pregnancy.
f. Do we have the STG and the ANC guideline in our working stations.
g. What happens when a woman has glucosuria, what is the next step.
h. What other tests/modalities do we do routinely to pregnant women to identify if she has diabetes
i. Among all the services, does a woman have to pay for rbg/fbg or urinalysis?

3. During our health education sessions that pregnant women are provided, what do we usually talk about?

Optional prompts for other members if not mentioned:

  a. Are health education session for pregnant women conducted
  b. If no, why are they not routinely done?are there challenges?
  c. Do you think we should do them? Why?
  d. How best can we re-institute the sessions
  e. Who creates the education topic
  f. Is there a reference for the selecting the topics
  g. Is diabetes during pregnancy among the topics
  h. Do we think it should be there

4. Now lets talk about the health care providers; what is the arrangement for receiving training in services that we provide at ANC?

Optional prompts for other members if not mentioned:

  a. What the common trainings offered
  b. Anyone trained on diabetes during pregnancy
  c. When was the last training on GDM conducted
  d. When is the next one offered?
  e. Do we think we know enough about diabetes in pregnancy compared to other conditions in pregnancy?

Version 1 (25/01/2024)

S1B appendix: Topic guide for IDI - Gynaecologist

1. Good morning, I am here to have a conversation about the services that a woman receives when she comes for ANC, particularly on screening for diabetes during pregnancy.
2. Can you tell me about yourself

   Optional prompts

   a. Educational background/level of training
   b. Years of work experience
   c. Position if any in the hospital/facility
3. Can you tell me how your week schedule is as a gynaecologist at the hospital

   Optional prompts

   a. Schedule for attending pregnant women during clinics (when do you see pregnant women coming for clinic).
   b. How does a pregnant woman come to you (ie self visit/referral, sent from the clinic by the nurses/MDs,)
   c. CME sessions on high-risk pregnancies for the juniors and nurses.
4. Now we will shift to women coming in for ANC visits, can you tell me, when a woman comes-in to the point of exit where and what she does on a routine clinic ANC visit.

Optional prompts for other members if not mentioned:

   i. Does she pay for any service? (cost sharing policy)
   j. How is she positioned to be attended (by you) in the order of her arrival to the clinic (cards collected/numbers given or what is done?
   k. Who is likely to see her first for a consultation upon arrival (doctor or nurse or both and what will make the difference)
   l. Which clients are shifted/relocated to be seen by you?

5. Then can we now talk about the screening modalities. In this facility, can you explain to me how you identify if a woman has diabetes during pregnancy.

Optional prompts for adding if not mentioned:

j. What is usually screened when a pregnant woman comes for a routine visit?
k. Do you screen for GDM? If yes – how and to what extent?
l. If no, can you explain why? (skip c if no)
m. When you have to order a blood sugar test/urine glucose for a pregnant woman, what happens until you get the results?
n. What are the challenges for you to screen for GDM at this facility – individual level (knowledge, skills, competency etc), facility level (work culture, supplies, attitudes, administrative support etc.) policies (training, available resources etc) others – planning, client challenges, no DM in this community.
o. What is available that makes you think will promote screening to be conducted routinely once started – ie willing staff, learning culture, space, leadership etc

If screening is done – ask the following probes

p. Which guideline/SOP are you using
q. What are the reasons to choose the modality we have chosen to use
r. If no screening done, why are we not doing?
s. Are you aware of Tanzanian guideline/s that are speaking about screening for diabetes in pregnancy
t. Do you have the STG and the ANC guideline in your working stations
u. What other tests/modalities do we do routinely to pregnant women to identify if she has diabetes
v. Among all the services, does a woman have to pay for rbg/fbg or urinalysis?

6. For ANC care, it is recommended to have health education sessions for attending clients. Can you talk to me the situation/status about the sessions here at the hospital? During our health education sessions that pregnant women are provided, what do we usually talk about?

Optional prompts for other members if not mentioned:

i. When (day of the week and time) is it done.
j. If not done, why?
k. Do we think it should be there
l. Why should we be doing the education sessions
m. How can we re-institute the sessions to be functional here. (skip f to h if not done)
n. Who creates the education topic
o. Is there a reference for selecting the topics
p. Is diabetes during pregnancy among the topics
q. What is your role in supporting building capacity to these nurses.

7. Now lets talk about the health care providers; what is the arrangement for receiving training in services that we provide at ANC?

Optional prompts for other members if not mentioned:

f. What are the common trainings offered
g. Anyone trained on diabetes during pregnancy
h. When was the last training on GDM conducted
i. When is the next one offered?
j. Do we think we know enough about diabetes in pregnancy compared to other conditions in pregnancy?
k. Do we conduct CMEs on maternal health in the facility, how do you support them.

8. We are coming to an end of our discussion, is there something that you feel I have missed and would want to talk about before we end our discussion.

Version 1 (25/01/2024)

S1C appendix: Topic guide for IDI – RCH clinic head/Matron + MDs

1. Good morning, I am here to have a conversation about the services that a woman receives when she comes for ANC, particularly on screening for diabetes during pregnancy.
2. Can you tell me about yourself

   Optional prompts

   d. Educational background/level of training

   e. Years of work experience

   f. Position if any in the hospital/facility
3. Can you tell me how your week schedule is as a head of RCH clinic at the hospital

   Optional prompts

   d. Schedule for attending pregnant women during clinics (when do you see pregnant women coming for clinic).

   e. CME sessions on high-risk pregnancies for other nurses.
4. Now we will shift to women coming in for ANC visits, can you tell me, when a woman comes-in to the point of exit where and what she does on a routine ANC clinic visit.

Optional prompts for other members if not mentioned:

m. Where does she register

n. What does she do after registration

o. The time that the clinic starts

p. Which service is offered first

q. Does she pay for any service? (cost sharing policy)

r. How is she positioned to be attended in the order of her arrival to the clinic (cards collected/numbers given or what is done?

s. Who is likely to see her for a consultation (doctor or nurse or both and what will make the difference)

t. How/where does she get a date for next visit

u. Average number of ANC attendees per day

v. Is there a separation for first attendees and followup

w. On average, at what gestation age do most women come for first ANC visit.

x. Type of laboratory tests that routine ANC attendees do at this facility

y. Is any of the laboratory tests paid for (If yes, which one).

5. Then can we now talk about the screening modalities. In this facility, can you explain to me how you identify if a woman has diabetes during pregnancy?

Optional prompts for adding if not mentioned:

w. Do you screen for GDM? If yes – how and to what extent?

x. If no, can you explain why? (skip c if no)

y. Can a nurse write/request for investigations to be done for a pregnant woman coming for ANC clinic?

z. When you have to order a blood sugar test/urine glucose for a pregnant woman, what happens until you get the results? (where is it requested, time to get the results, do you always get the results etc).

aa. What are the challenges for you to screen for GDM at this facility – individual level (knowledge, skills, competency etc), facility level (work culture, supplies, attitudes, administrative support etc.) policies (training, available resources etc) others – planning, client challenges, no DM in this community.

bb. What is available that makes you think will promote screening to be conducted routinely once started – ie willing staff, learning culture, space, leadership etc

If screening is done – ask the following probes

cc. Which guideline/SOP are you using

dd. What are the reasons to choose the modality we have chosen to use

ee. If no screening done, why are we not doing?

ff. Are you aware of Tanzanian guideline/s that are speaking about screening for diabetes in pregnancy

gg. Do you have the STG and the ANC guideline in your working stations

hh. What happens when a woman has glucosuria, what is the next step

ii. What other tests/modalities do we do routinely to pregnant women to identify if she has diabetes

jj. Among all the services, does a woman have to pay for rbg/fbg or urinalysis?

6. We will now switch to health education sessions at our ANC clinic. For ANC care, it is recommended to have health education sessions for attending clients. Can you talk to me the situation/status about the sessions here at the hospital?

Optional prompts for other members if not mentioned:

r. Are health education sessions done?

s. During our health education sessions that pregnant women are provided, what do we usually talk about?

t. When (day of the week and time) is it done.

u. If not done, why?

v. Do we think it should be there

w. Why should we be doing the education sessions

x. How can we re-institute the sessions to be functional here. (skip f to h if not done)

y. Who creates the education topic

z. Is there a reference for selecting the topics

aa. Is diabetes during pregnancy among the topics, if not, should it be included? How can it be included

7. Now lets talk about the health care providers; what is the arrangement for receiving training in services that we provide at ANC?

Optional prompts for other members if not mentioned:

l. What are the common trainings offered
m. Anyone trained on diabetes during pregnancy
n. When was the last training on GDM conducted
o. When is the next one offered?
p. Do you think we know enough about diabetes during pregnancy compared to other conditions in pregnancy?

8. We are coming to an end of our discussion, is there something that you feel I have missed and would want to talk about before we end our discussion.

Version 1 (25/01/2024)

S1D appendix: Topic guide for IDI – Laboratory manager/practitioner

1. Good morning, I am here to have a conversation about the services that a woman receives when she comes for ANC, particularly on screening for diabetes during pregnancy.
2. Can you tell me about yourself

   Optional prompts

   g. Educational background/level of training

   h. Years of work experience

   i. Position if any in the hospital/facility
3. Can you tell me how your week schedule is as a head of laboratory at the hospital

   Optional prompts

   f. Are there days when there are more pregnant women at the laboratory

   g. Are pregnant women given priority for processing samples

   h. Are there CME sessions conducted to the junior departmental members
4. Now we will shift to women coming in for ANC visits, can you tell me, the process a woman takes until she arrives to the laboratory for tests.

   Optional prompts if not mentioned:

   z. Can she come directly here

   aa. Is there a documentation that she comes with, what type of documentation

   bb. How are laboratory test requests sent to the lab (Are requests sent electronically?)

   cc. Are the tests for pregnant woman charged? Which ones

   dd. How do you submit the results

   ee. What are the common laboratory tests you receive from ANC.

5. Then can we now talk about the tests. In this facility, can you explain to me how the situation is on availability of supplies for testing blood glucose and urine glucose.

   Optional prompts for adding if not mentioned:

kk. Do you have rapid test kits? Which ones

ll. Are the test kits functional

mm. In the past 3 months, have you run out of stock for the test kits

nn. Frequency you receive requests for FBG/RBG or urine glucose from ANC (for pregnant women)

oo. Are there SOPs for processing these samples? Where are they.

6. What are the challenges for you to do blood or urine glucose tests at this facility – individual level (knowledge, skills, competency etc), facility level (work culture, supplies, attitudes, administrative support etc.) policies (training, available resources etc) others – planning, client challenges, no DM in this community.
7. We are coming to an end of our discussion, is there something that you feel I have missed and would want to talk about before we end our discussion.
