# Supplementary material for: “We have theoretical knowledge, but these are not things we do regularly”: District hospital’s healthcare workers’ experiences and perceptions on gestational diabetes mellitus screening in Tanzania
Source: PLOS Glob Public Health. 2025 Nov 7;5(11):e0005373. doi: 10.1371/journal.pgph.0005373 (PMC12594363; doi:10.1371/journal.pgph.0005373)
Supplement: S2 Text — (PDF) [file pgph.0005373.s002.pdf]

Version 1 (24/04/2023)

Optimizing screening practice for gestational diabetes mellitus in primary healthcare facilities in Tanzania

S2 Appendix: Assessment Checklist

1. Name of the facility
   a. Mbagala rangi tatu District hospital
   b. Kisarawe District hospital
2. Date of assessment / / / (date/month/year)
3. Time of start of the assessment ______________
4. Fill in the following table accordingly.

Item	Availability (YES/NO)	Where was it located (Nurses' office – a; Doctors office – b; MOIs office – c; Matrons office – d; other – e (mention____________) Not applicable – f.	Comment
**Antenatal clinic**			
Antenatal care guideline (if available, comment which version is it – year of publication)			

Standard treatment guideline			
Posters or education materials for education on GDM			
SOP/Protocol for screening GDM			
A checklist for risk scoring of women			
A topic on GDM on the monthly educational material topics			
Weighing scale (comment if functional and when was it calibrated)			
Height board (comment if functional)			
Blood pressure machine (comment if its functional and how many they are at ANC clinic)			

Total number of staff at ANC per cadre and years of work			
Total number of staff per shift per cadre and years of work			
Number of shifts per cadres			
**Laboratory**			
Clean gloves			
Sterile gloves			
Alcohol swabs			
Lancet prickers			
Biochemistry analyzer machine			
Reagents for biochemistry analyzer machine			
Glucometer (comment on the type of machine, functionality, and strips availability)			
Urinalysis test strips			

Glucose load (75 gm) for OGTT			
SOP for urine testing (comment on its location)			
SOP for glucose testing (comment on its location)			
Total number of staff per cadre and years of work			
Total number of staff per shift per cadre and years of work			
Number of shifts			

5. Do women pay for screening of urinalysis - glucosuria (Matron in-charge)
   a. YES
   b. NO
   c. I don’t know
6. If yes in 4, how much _______________ Tsh.
7. Do women pay for screening of blood glucose test (Matron in-charge)
   d. YES
   e. NO
   f. I don’t know
8. If yes in 4, how much _______________ Tsh.
9. In the past three months, have providers received a refresher training on GDM at the ANC?

a. YES

b. NO

c. I don’t know

10. If YES in 9, how many ___________

11. If YES in 9, which cadre ___________

12. If NO in 9, is there a scheduled training in the next three months on GDM

    a. YES

    b. NO

    c. I don’t know.
