# Supplementary material for: “We have theoretical knowledge, but these are not things we do regularly”: District hospital’s healthcare workers’ experiences and perceptions on gestational diabetes mellitus screening in Tanzania
Source: PLOS Glob Public Health. 2025 Nov 7;5(11):e0005373. doi: 10.1371/journal.pgph.0005373 (PMC12594363; doi:10.1371/journal.pgph.0005373)
Supplement: S3 Text — (PDF) [file pgph.0005373.s003.pdf]

Version 1 (24/04/2023)

Optimizing screening practice for gestational diabetes mellitus in primary healthcare facilities in Tanzania

S3 Appendix: Facility Structured observation guide

1. What is there:
    a. Describe what is there to support health education, screening/testing for GDM.
        - Waiting area – enough seating space to accommodate every woman/couple present, is there a shade, is the person giving health education audible for the whole group, time used for providing health education.
        - Consultation room – is the space enough for a couple.
        - Notice board with flow charts or educational materials – general and relevant to GDM.
        - Registration place – distance from the ANC room/consultation room
        - Access to the guidelines and SOPs (relevant to GDM) – Are they present within the consultation rooms.
        - Health Education provision guide – if there is a topic for GDM.
        - Laboratory – SOP for testing and results, supplies for testing, disposal of the wastes.
        - Are there urine strips for urinalysis.
        - Is there a blood glucose machine? Is it working?
        - Do they take stock for available strips (urinalysis and blood glucose) if will be enough for the day.
2. Who is there; Describe the following
    a. Number of health care providers per shift (per task performed – health education, consultation, laboratory, other logistics)
    b. When do they exchange shifts?

   c. Are there males (partners of the attending women) in attendance?
   d. Number of patients per time (morning vs afternoon)
   e. When does the clinic end
   f. Who is doing the testing (blood test and urine test)

3. What is happening; Describe the following
   a. Flow of women from arrival to exit – where do they start to the point they end (From entry to leaving)
   b. What is being communicated during the health education sessions – Is GDM part of the topics
   c. How women and health care providers interact
4. Time for consultation (time a woman spends in the consultation room)
